# Whole Life: a feasibility study of a recovery-focussed intervention in patients with stabilised schizophrenia

**DOI:** 10.3310/nihropenres.13220.2

**Published:** 2022-02-14

**Authors:** Tim M. Gale, Jan Woodward, Glynis Meredith-Windle, Thanusha Balakumar, Brian Littlechild, Chris J. Hawley

**Affiliations:** 1 Department of Research, Hertfordshire Partnership University NHS Foundation Trust, Hatfield, AL10 8YE, UK; 2 School of Life & Medical Sciences, University of Hertfordshire, Hatfield, AL10 9AB, UK; 3 School of Health & Social Care, University of Hertfordshire, Hatfield, AL10 9AB, UK; 4 ArcHealth, St Albans, UK; 5 Hertfordshire Partnership University NHS Foundation Trust, Hatfield, AL10 8YE, UK

**Keywords:** Recovery, Social Adaptation, Co-production, Feasibility, Psychological, Intervention, Whole-Life, Schizophrenia, Mental Health

## Abstract

**Background:**

The Recovery Approach is about supporting people to live the best life they possibly can. This paper reports on a 2008–11 study of a recovery-focussed, one-to-one coaching programme called Whole Life (WL) in a group of people with stabilised schizophrenia. WL comprises 15 modules, each addressing an aspect of life that may pose challenges for someone with mental illness. It involves regular meetings with a coach, additional homework activities and lasts approximately one-year. This level of commitment requires participants to be motivated and enthusiastic.

**Methods:**

This was a non-randomised feasibility study, designed to assess acceptability and potential benefits of WL. The WL group was compared to another group of people with the same diagnosis, who received their usual treatment. This was not a strict control group. The primary outcome measure was the Social Adaptation Self-Assessment Scale.

**Results:**

Of those recruited to the WL group, 33/44 (75%) completed the full programme. WL participants showed an 11-point increase in mean SASS between baseline and Week 60. Subjective ratings showed benefits of WL at 3 and 6 months after the intervention had ceased, with most saying they felt better and none saying that they felt worse. The comparison group was more ill than the WL group at baseline and showed some improvement over the course of the study, albeit at a lower level than the WL group. However, controlling for baseline group differences meant that none of the outcome measures could reliably distinguish between WL and comparison groups.

**Conclusions:**

The study showed that WL is an acceptable and helpful intervention for motivated and enthusiastic individuals. It may have wider applicability for people with a less serious and chronic mental illness, although we do not know how it compares to other interventions. We discuss some methodological limitations of the study.

## Plain language summary

*Recovery* is about helping someone to live the best life possible, even though they may have a mental-illness. Whole Life (WL) is a 15-module coaching programme, based on principles of Recovery. A WL coach is a mental-health support-worker who meets regularly with a participant, and they use the WL Manual to help make positive changes.

We worked with a group of coaches and participants to test the WL programme. The participants all had a diagnosis of schizophrenia. We wanted to know how acceptable participants found the WL programme, and whether it made a difference to them. Three-quarters of people who started the WL programme completed it. Those who dropped out found it difficult to meet their coach regularly, and to complete the homework. But those who completed WL found it helpful, saying they felt better than before they started. We also asked participants to complete some assessment scales. The main scale looked at friends, social activities and hobbies. We also used a scale to measure anxiety and depression. The people who completed WL felt less anxious and depressed, and were more socially active, than before they began WL.

We also followed another group of people with schizophrenia, who were not offered WL. This group was more ill than the Whole Life group. We adjusted for this when comparing the two groups and found that the comparison group also improved during the course of the study. Some people may have had other therapies that we did not know about. It is therefore difficult to say whether WL is effective for everyone or better than other treatments.

We think WL is probably best suited to younger people who don’t experience many changes in their symptoms. WL requires commitment and enthusiasm, but can lead to helpful improvements in activity, mood and social life.

## Introduction

### Recovery approach in mental health care

It has been argued that mental health services sometimes fail those with severe illness by lacking orientation towards the single most important goal: recovery ( Farkas, 2007). Over the last few decades, social care and mental health services have become much more oriented towards ‘recovery’ based models of care ( Department of Health, 2011; Lieberman *et al*., 2008; McCabe *et al.,* 2018; SCIE, 2007; Shepherd *et al*., 2008; Slade, 2009; Slade *et al*., 2014; Slade & Longden, 2015; Slade *et al*., 2017; SLAM & SWLSTG, 2010; Warner, 2009). The aims of the recovery approach are concisely encapsulated by Townsend & Glasser (2003) as being ‘about treating the whole person, identifying their strengths, instilling hope, and helping them to function at an optimal level, by allowing them to take responsibility for their life’. Some general aspirations of the recovery approach are that people should: (i) have reduced dependency on the mental healthcare system, being more able to identify and manage their own health-care and social-care needs, (ii) be economically active, pursue education and have less dependency on state benefits, (iii) have meaningful social networks and relationships that are independent of psychiatric or social services, (iv) re-capture a more purposeful and fulfilling existence. Of course personal recovery may mean very different things to different people, but the essence of the approach is about supporting people to live the best life they possibly can. One of the main criticisms of the recovery approach is that it lacks objectivity and is therefore open to misuse, especially where outcome measures are concerned. For example, two individuals who are said to be ‘recovered’ may have completely different underlying mental illnesses. If ‘recovery’ is taken as the key outcome, there is concern that, for example, this could lead to people being prematurely discharged from treatment services. The recovery approach is not then an alternative to medical models of treatment, but is helpful when used in conjunction, taking a more holistic approach.

### The Whole Life programme

The Whole Life (WL) programme is a recovery-oriented 15-module life-coaching manual, conceived and assembled by one of the co-authors (JW), for use in one-to-one sessions between a mental health support worker and a service-user. The modules include: (1) Dreams and aspirations; (2) Your journey – choose the destinations; (3) Physical environment; (4) Communication skills; (5) Assertiveness; (6) Self-esteem; (7) Action-planning; (8) Managing time, change and disappointment; (9) Health, anxiety and stress; (10) Physical health; (11) Understanding and managing money; (12) Examining beliefs and values; (13) Pathways to education and work; (14) It’s OK to have fun - leisure and recreation; (15a) Personal relationships; (15b) Closing session for programme end. A more detailed breakdown of the 15 modules is included in the *Extended data* ( Gale, 2021), along with a brief introduction for coaches.

For some people, having a mental illness can cause them to doubt their own abilities and to lose confidence in themselves and their future. Their illness may have been so overwhelming that they begin to identify as a patient more than a person, with all focus being placed on managing illness and symptoms. WL aims to restore a more healthy sense of self, and to move the participant to a position where they are able to take greater control of their life. The WL programme starts with the participant’s self-assessment of (i) where they are currently, (ii) where they would like to be, and (iii) what support they need to help them realise their aspirations. WL does not seek to cure mental illness, nor eradicate symptoms, but aims to empower participants to focus and build on strengths, and to develop greater resilience (following Rapp & Goscha, 2006).

WL is best thought of as a flexible coaching programme to be used jointly between a mental health professional (the coach) and service user (the participant). Each module focuses on a particular area of life that may be difficult for a person with chronically poor or unstable mental health, and for which a level of personal recovery should be achievable. The modules are structured to include: (i) some briefing notes for the coach, which summarise the approach for the module in question, (ii) some questionnaires and graphical materials for helping to explore the participant’s self-perception, and (iii) some resource pages, used by coach and participant to provide a basis for their work. There is an expectation that the participant will engage in homework tasks between sessions, and these may include self-reflection, practicing what has been learned, and setting personal goals. WL has no fixed duration, nor pace of work; rather, the duration and intensity is adapted to suit the individual participant. However, the programme typically lasts about a year, with the coach and participant meeting weekly or bi-weekly. Meetings can take place wherever the participant is most comfortable (e.g., own home, public space, coffee shop, private room in a community hub), and the meeting location can be varied to help support the participant in expanding their comfort zone over time. A core feature of the WL programme is that the mental health professional should try to stand aside from a position of knowledge and/or authority and aim to facilitate and support the participant towards finding solutions or improvements, rather than delivering or prescribing them. The WL approach draws strongly from the theory of Learned Helplessness ( Maier & Seligman, 2016; Seligman, 1975; Seligman, 2002; Seligman *et al*., 2005), which posits that when an organism or individual lacks control over an unpleasant environment or circumstance, the resulting feeling of helplessness may become permanent, thereby reducing an individual’s capacity to find a way out. Once learned helplessness has set in, an individual may become resistant to change, even if an opportunity to escape is offered. People with long-term mental health conditions frequently experience learned helplessness ( Maier & Seligman, 2016) and may need support to give them back a feeling of empowerment. Whole Life recognizes this and aims to address it by restoring confidence and control across a broad range of life issues. Coaches and participants should seek to find a balance that maintains a safe place for the participant, but not so safe that the desire to make changes (i.e. escape from a feeling of helplessness) is suffocated. The participant should always be in control, taking responsibility for their own wellness and setting the pace of progress and travel themselves, rather than abdicating it to professionals to bring about recovery. Participants are encouraged to put what they have learned and discovered to practical use, in their day-to-day life. Apart from the introductory and final modules, there is no prescribed order: participants can choose to spend more time on those modules that are most relevant. The key mechanism of effect is supporting the individual to feel in control of their life and future. The way in which this will happen will, of course, differ from person to person.

WL was not conceived nor designed for a specific mental health diagnosis. Rather it is a programme that can be used by anyone who is currently receiving mental healthcare, and who wants to make changes in their life. The depth of focus and amount of time allocated to each of the modules is very much driven by the participant. Prior to undertaking this research study, we had carried out some pilot work on the WL programme with people who had a broad range of psychiatric diagnoses and associated life difficulties. This work suggested that motivated participants could make significant gains using WL even if they still had clinically significant symptoms. The most notable improvements were seen in social functioning, and it also became evident that motivation, commitment and a willingness to change, were absolutely key to participants remaining in, and benefitting from, the WL programme. Prior to this study, WL had not been subjected to formal research testing, and so this is the first study to report on its feasibility, acceptability and potential benefits. Although WL was designed as a widely applicable approach, it would be unconventional to have a novel intervention tested within a very heterogenous population. For this reason, we decided to focus the study on people with a diagnosis of schizophrenia.

### Schizophrenia

Schizophrenia is a chronic and severe psychiatric disorder affecting up to 20 million people worldwide ( WHO, 2019). Lifetime prevalence is 0.3% to 0.7% ( Javitt, 2014), and is 2–3 times more common in males ( Abel *et al*., 2010; Iacono & Beiser, 1992). It is characterised by abnormalities in thought, perception and behaviour, often experienced as hallucinations (most usually, hearing voices), delusions and disordered thinking – the so called ‘positive’ symptoms. Longer-term ‘negative’ effects, even after treatment, may include social withdrawal, loss of affect, paranoia and cognitive impairment. It has long been viewed as a heterogenous disorder ( Blueler, 1911), with significant variation in symptomatology from person to person. People with schizophrenia are known to have premature mortality ( Hoang *et al*., 2011; Laursen *et al*., 2011; Laursen *et al*., 2014; Saha *et al*., 2007), and have been less likely to receive a diagnosis and treatment for cardiovascular conditions than people without schizophrenia ( Smith *et al*., 2013), despite elevated rates of smoking ( Castle *et al*., 2019; Ziaaddini *et al*., 2009) and obesity ( Holt & Peveler, 2009; Whicher *et al*., 2019). Historically, long-term outcomes have been poor ( Jobe & Harrow, 2005). For example, Harrison *et al*. (2001) found, in a long-term follow up study, that 11.6% cases were in continual institutional care, while approximately half were continuously symptomatic, a half were unable to sustain employment and only 16–38% were in a state that could be consider to be ‘recovered’ depending upon the criteria that were applied to define recovery.

Many people who have been diagnosed with schizophrenia have poor employment prospects, may not enjoy the benefits of relationships and sexual engagement ( Smith *et al*., 2002), have limited aspirations, minimal social networks, and less engagement in society than they might ideally choose. Given these difficulties, it is perhaps unsurprising that suicide rates have been high in this population ( Hor & Taylor, 2010; Ösby *et al*. 2000). Although positive symptoms can often be managed using antipsychotic medication ( Leucht *et al*. 2003; Tandon & Jibson, 2003), impairments of a more social and functional nature can be chronic. People who have schizophrenia are often cautious about leaving their comfort or buffer zone ( Mortensen *et al.,* 2016; Sparrowhawk, 2009) because they fear that the ensuing stress may trigger another episode. This can leave them socially withdrawn and lonely. Although some psychological therapies such as cognitive behavioural therapy (CBT) have been proposed to deliver improvements in mood and cognition, the effect sizes are small, highly variable and have tended to be short-lived ( Laws *et al*., 2018; Lynch *et al*., 2010; Morrison *et al*., 2018; Wykes *et al*., 2008). Early intervention offers the best chance of recovery, and this is now central to the treatment of schizophrenia in the UK ( NICE, 2016) and indeed other parts of the world. How to maintain the benefits of Early Intervention in Psychosis services, post discharge, is one of the key themes that has been previously identified for future research ( NICE, 2014), and there is current emphasis on developing and researching more tailored interventions for people with schizophrenia ( NICE, 2020).

### Co-production

This study had a significant level of co-production from the outset. The WL manual itself had been refined over many years, based on the feedback and experiences of service users who had previously engaged with the programme. The module structure had evolved over the course of several years, in response to specific needs that were expressed by community mental health teams and their service users. The term ‘Whole Life’ was chosen to reflect that the manual seeks to cover a very broad range of life issues that pose challenges for people with enduring mental illness. Therefore, the manual itself, had a strong element of co-production and evolution, which was distinct from the patient and public involvement in the feasibility study described here. Some of the people who had previously been recipients of Whole Life were invited to join the research team in a half-day session, prior to the grant submission, where we discussed the research proposal and invited them to contribute their lived experience to the design and planning of the study. We specifically asked the group to help us identify appropriate outcome measures that they thought would best capture the benefits. The group also advised on study design, suggesting that randomisation could be problematic and would likely lead to a high dropout rate in a control or comparison group, primarily because there would likely be disappointment for those people excluded from the active intervention. And there would then be a wait of at least one year before control group participants could receive the active intervention. For this reason, we developed the original funding application as a single arm study.

### Study aims

The primary aim of the study was to assess the feasibility of recruiting people with a diagnosis of schizophrenia to a year-long, recovery-focussed, one-to-one coaching programme using the WL manual. As part of our feasibility assessment, we wanted to know the following:

i. What is the level of uptake of WL?
ii. Of those who sign up to WL, how many stick the programme?
iii. Would any of them feel any better on account of taking part?
iv. Which instruments are best suited for measuring change in the area of interest?
v. How would mental health professionals adapt to being WL coaches?

As noted previously, we had originally intended this to be a single-arm feasibility study, given that there was no existing published evidence in support of WL. However, the NIHR and reviewers were keen for us to include a treatment-as-usual or alternative therapy comparison group. Given that funding was limited, and that this study preceded the current system for excess treatment cost resourcing, we were unable to offer an alternative therapy group. We therefore opted to collect relevant outcome measure data from people with the same diagnosis in other NHS Trusts, where patients would not be expecting to receive the WL intervention but would be receiving their usual treatment.

Aside from feasibility then, we also set out to measure whether WL led to any measurable and sustained improvements in a cohort of patients with stabilised schizophrenia, relative to a group who did not receive WL. The primary outcome measure for this was the Social Adaption Self Assessment Scale (SASS; Bosc *et al*., 1997). This scale assesses a number of domains that are often within the focus of Recovery-based approaches (e.g. community life, quality of spare time, relationships), and was selected as the primary outcome measure by our co-production group. Although the SASS is widely used in the depression literature, it has also been successfully used to measure social function in people with schizophrenia ( Khamina *et al.,* 2021; Vyas *et al.,* 2007). Secondary aims were to investigate whether WL led to reductions in anxiety and depression levels, led to reduced schizophrenia symptomatology, as well as bringing about improvements in occupational functioning and other more general aspects of daily life.

## Methods

### Design

This was a non-randomised, non-blinded, feasibility study of the WL intervention in people with stabilised schizophrenia (see participant inclusion and exclusion for clarification about ‘stability’). It was run from October 2008 until March 2011. Suitable participants in Hertfordshire Partnership University NHS Trust were offered WL and were paired with a mental health professional for up to one year, with the expectation that they would meet with their coach on a weekly, or bi-weekly, basis. Participants were expected to complete the study outcome measures at baseline, week 24, week 48, week 60 and week 72. Week 60 was regarded as the primary study endpoint (approx. 3 months post-intervention), with week 72 offering a longer-term follow up. A non-randomised, comparison group was recruited from three other mental health trusts in London and Essex. This group did not receive any WL coaching, but participants consented to be assessed on the primary outcome measure at all time points and on all screening and secondary outcome measures at baseline and endpoint (week 60). Although this group cannot be regarded as a control group, it offered the most feasible and affordable means of providing a treatment-as-usual comparison to the WL group at the time this study was run. The study was not registered on any clinical trials databases.

### Participants: inclusion and exclusion

Participants in the WL group were patients aged between 18 and 65 years, who had received a diagnosis of schizophrenia, delusional disorder, schizoaffective or schizophreniform disorder, and were stabilised on, and compliant with, anti-psychotic medication. A judgement about diagnosis, symptom stability and medication compliance was made by the most senior treating clinician for each participant. Anyone who was estimated to be less than 80% compliant with the prescribed dose of existing medication was not included, and nor was anyone whose symptom profile was prone to substantial weekly or monthly fluctuations. Patients who had been hospitalised within the previous month, or were subject to any legal restriction (court order, probation or Mental Health Act), were also excluded. Occasional cannabis or alcohol use was not an exclusion criteria, but a history of long-term substance abuse or alcohol dependency was. Other exclusion criteria were meeting DSM-IV criteria for affective psychosis or borderline personality disorder, previous treatment with WL for more than 30 days, and currently taking higher than usual doses of antipsychotics, either as monotherapy or in combination. All included participants were required to have a score of 35 or lower on the SASS, at baseline. The normative UK mean score on the SASS is approximately 42.4 ± 6.4 ( Jefferies & Gale, 2011), so all participants scored at least one SD lower than the UK average, with many scoring 2 or more SDs lower. Scores on any other screening measures or secondary outcome measures (see *Data collection and outcome measures* section) were neither inclusionary nor exclusionary.

Participants were required to continue taking their existing medication and continued to receive any usual treatment from their clinical team. However, any patients who were awaiting, or currently receiving, any form of structured psychotherapy (including CBT, interpersonal therapy, cognitive-analytic therapy and psychodynamic therapy) were excluded from taking part. This step was taken (i) to reduce the likelihood that any observed improvements in outcome measures were attributable to a different therapeutic intervention and (ii) to ensure that existing or planned psychological interventions were not interrupted by the recipient taking part in this research study.

Participants in the comparison group fulfilled the same inclusion and exclusion criteria, except that restrictions were not placed on whether they could receive psychological interventions.

All participants in the WL group were first approached about the study by their clinical teams and, if interested, they received a follow-up call and invitation to a meeting with one of the study team. All participants were provided with written information about WL and the research study, and were given at least 24 hours to consider this before consenting to take part. It was emphasized that enrolment in the WL programme would require an ongoing commitment from the participant to attend regular meetings with their WL coach and to be willing to undertake some homework exercises in between meetings. Participants who were thought unlikely or unable to commit were not invited to take part. Consent was taken by a member of the research team in Hertfordshire, or by a Mental Health Research Network (MHRN) representative in the other Trusts. This study was run between 2008 and 2011 and preceded the HRA approval system. Ethics approval was given by the Hertfordshire Ethics Committee (08/H0311/122) and R&D approval was obtained from each participating NHS Trust.

Written informed consent for participation in this study was given by all service users who took part. No personal data is discussed in this paper.

### Participants: coaches

Two NHS mental health support workers were employed on the study, and each coached 10 participants. The remainder of the coaches were existing NHS staff (mainly support workers, but also psychology assistants, nurses and a social worker) who each worked with one or two service users within their existing caseload. All coaches received the same whole-day training package run by the WL manual author (JW), which also included vignettes involving previously experienced WL coaches. All coaches received monthly supervision and updates from JW and one of the experienced coaches, throughout their time spent in the study. This helped to reduce variability in approaches, and ensured that all coaches were able to discuss concerns and share learning on a regular basis. Each coach and each participant received a hard-bound copy of the WL manual, which was theirs to keep and make notes in. Coaches and participants were matched primarily on a geographical basis: we sought to make it as easy as possible for the regular meetings to go ahead. Participants in the comparison group were not offered the WL intervention. However, the three participating NHS Trusts who supported the comparison (treatment as usual) arm were offered access to the WL manual and associated training once data collection was complete.

### Data collection and outcome measures

All participants were assessed at baseline (week 0), and weeks 24, 48, 60 and 72. The week 60 and 72 assessments were short to medium-term follow-up measures, falling approximately 3 and 6 months after the intervention had ended. Week 60 was considered the primary study endpoint because we wanted the key outcome measurement to happen once regular contact with the coach had stopped. Data collection generally occurred within a private room in an outpatient clinic or community mental health team hub. The Positive and Negative Symptoms Scale (PANSS; Kay *et al*., 1987) was used at baseline to help characterize the degree of symptomatology in the sample and to determine whether active (WL) and comparison groups were broadly similar. The primary outcome measure was the SASS ( Bosc *et al*., 1997), which was collected at all assessment intervals. Levels of anxiety and depression were measured using the self-rated Hospital Anxiety and Depression Scale (HADS; Snaith, 2003). Schizophrenia symptom severity was succinctly measured by the Clinical Global Impression for Schizophrenia Scale (CGI-S; Haro *et al*., 2003), and occupational function was recorded using the Social and Occupational Function Scale (SOFAS; Morosini *et al*., 2000). Some of the more general aspects of daily life were captured using the Health of the Nation Outcome Scale (HoNoS; Wing *et al*., 1996). All these outcome measures had been selected by our co-production group, as being broadly compatible with the general aims of Recovery approaches. The HADS, SOFAS, HoNOS and CGI Schizophrenia Severity scale were all designated as secondary outcome measures and were completed at baseline and week 60.

A Perception of Impact of Therapy (PIT) questionnaire was also completed by the WL participants at weeks 48, 60 and 72. The PIT questionnaire was a 7-point, self-rated, scale which asked the participant to compare how they felt at the current time of rating, compared with how they had felt before they began the coaching programme. The scale intervals are as follows: 1 - I am very much worse, 2 – I am much worse, 3. I am slightly worse, 4 - I am neither better nor worse, 5 – I am slightly better, 6 - I am much better and 7 - I am very much better. Comparison group participants did not complete the PIT as it did not apply to them. All research assessments were carried out by dedicated clinical research staff, although these assessments were sometimes scheduled prior to a coaching session for the participant’s convenience. Where possible we ensured that the ratings for a given participant across the study were always collected by the same clinical researcher. Although some of the outcome measures are self-rated, the clinical researcher could assist, discuss and clarify any of the scale items, and check those measures for any missed items. The clinical researcher also completed the clinician-rated outcome measures. Outcome measures for participants recruited to the Comparison Group were collected by MHRN staff. All research staff involved in this study were appropriately trained in the relevant scale administration and received study training from one of the lead investigators.

A qualitative module was also run but the results are not reported in this paper. The qualitative module was co-produced and involved interviews with participants who had dropped out from having WL and a random sample of those participants who completed WL. The results of this study are published elsewhere ( Littlechild *et al*., 2014).

### Sample size

For an assessment of feasibility and acceptability, we originally aimed to recruit 25 people to the WL intervention. However, in order to reference any recorded changes in outcome measures to the comparison group, we re-estimated study sample size based on a predicted 5-point difference in the primary outcome measure (SASS) between WL and comparison groups at week 60. An alpha and beta level of 0.05 and 0.2, respectively, yielded a sample size of 33 participants in the WL group and 50 in the comparison group. The higher number in the comparison group allowed us a more affordable means of achieving statistical power and meeting target recruitment. We allowed for a 25% dropout rate in both groups, so aimed to recruit 44 WL participants and 67 comparison participants.

### Statistical analysis

This paper presents quantitative analyses only. Feasibility data on the WL group (recruitment and retention, compliance with measure reporting, perception of benefit and retention of coaches) is largely presented as descriptive data. Significant baseline differences between WL and comparison groups were determined using unpaired *t* tests for continuous variables and Chi-square tests for categorical variables. Given that we could not ensure group matching on the various screening and outcome measures at baseline, any between-group comparisons were analysed using ANCOVA models, with baseline score, and any other measures that had exhibited significant group differences at screening/baseline, entered as co-variates. All statistical analyses for this study was carried out using JASP v0.14.1.

## Results

### Recruitment and retention

A total of 61 people were screened for the WL group, from which 44 participants were consented. Of these, 33 completed the study (75%), while 10 formally withdrew and 1 was lost to follow up. In the comparison group, 86 were screened of which 76 consented. However, of these, only 46 (61%) completed all the study assessments, 8 formally withdrew and 22 were lost to follow up. All analyses presented in this paper are based on completers ( *n* = 79) only.

The main reason for withdrawal in the WL group is that some participants were simply unable to commit to the regular schedule of meeting with the coach and doing the homework exercises in between meetings. When more than one month had elapsed, participants were asked if they wished to continue or withdraw from the study. It was our experience that most participants who let a month or more elapse between sessions did not wish to continue, although they were permitted to continue if they still wished. One participant moved away from the area and could not be followed up. Among the 33 who completed WL, adherence to the programme was very good. However, it did require coaches to be flexible about when and where they could meet their participants.

We recorded the number of hours of coach and participant contact across the whole study and this averaged 32.3 (± 11.4, range 13.5-59.9) hours. We also recorded the additional time spent by coaches in preparing materials and travel, and this averaged 23.7 (± 15.8, range 3.6-55.3) hours per WL participant. In practice, there were some weeks where no contact occurred due to holiday, leave or sickness. Across all WL completers, there was a mean of 29.9 (±9.2, range 13-51) face-to-face meetings, so we can infer that the typical participant had approximately 30 meetings with their coach, each lasting just over an hour. A key feature of WL is that it permits flexibility, so we did not set a minimum level of engagement. Some participants chose longer but less frequent meetings, while others preferred to meet every week but for a shorter time.

In the comparison group, 8 participants formally withdrew because they did not wish to undertake any further research assessments. A further 22 were lost to follow up, for a variety of reasons, including failure to respond and moving away.

### Compliance with outcome measures

Table 1 shows compliance with outcome measure recording for the two groups. The level of compliance was high for both groups, though dropped off slightly at weeks 24 and 48. This does of course reflect that we are only reporting here on study completers, but does show that even with dedicated research staff doing the rating collection, there was still a risk of outcome data going unrecorded.

**Table 1.** Percentages of outcome measures completed fully for each group across the study duration (completers only). Social Adaptation Self-Assessment Scale (SASS), Social and Occupational Function Scale (SOFAS), Hospital Anxiety and Depression Scale (HADS), Health of the Nation Outcome Scale (HoNOS), Clinical Global Impression of Schizophrenia (CGI-S), and Perception of Impact of Therapy (PIT).

	Whole Life group ( *n* = 33)					Comparison group ( *n* = 46)				
*Week*	0	24	48	60	72	0	24	48	60	72
SASS	100%	85%	85%	100%	100%	100%	85%	98%	98%	98%
SOFAS	100%	N/A	N/A	100%	N/A	98%	N/A	N/A	98%	N/A
HADS	100%	N/A	N/A	100%	N/A	100%	N/A	N/A	98%	N/A
HoNOS	100%	N/A	N/A	100%	N/A	100%	N/A	N/A	98%	N/A
CGI-	100%	N/A	N/A	100%	N/A	98%	N/A	N/A	98%	N/A
PIT	N/A	N/A	88%	100%	100%	N/A	N/A	N/A	N/A	N/A

Participants generally found the two self-rated assessments (SASS and HADS) easy to complete. A researcher was present at weeks 0 and 60 to complete the HoNOS, SOFAS and CGI(schiz) and was able to ensure full and proper completion of the two self-rated scales also.

### Perception of impact of WL

All those who completed the WL programme were asked (at weeks 48, 60 and 72) to make an assessment of how they felt as a result of their participation in the WL programme, compared with how they felt before starting it. No participants said that they felt worse, and most reported an improvement, with a quarter to one-third indicating that they felt very much improved. This data is shown in Table 2.

**Table 2.** Ratings given for impact of Whole Life intervention at weeks 48, 60 and 72, using a PIT scale. A score of 1 indicates ‘I am very much worse’, a score of 4 indicates ‘I am no better and no worse’ and a score of 7 indicates ‘I am very much improved’.

*Rating*	*1*	*2*	*3*	*4*	*5*	*6*	*7*	*n*
*Week 48*	0	0	0	2	8	9	10	29
*Week 60*	0	0	0	4	9	12	8	33
*Week 72*	0	0	0	2	9	13	9	33

### Coaches

WL coaches were all mental health professionals from Hertfordshire Partnership University NHS Trust. A total of 24 were recruited, including 2 support workers that were specifically employed on the study. The other 22 comprised support workers (14), nurses (2), social workers (2) and psychology assistants (4). No coaches withdrew from their role and there was a consistently high uptake of supervision.

### Between groups comparisons

There was no randomisation to WL and comparison groups and no a-priori matching between groups at baseline. The baseline characteristics of the WL and comparison groups are shown in Table 3.

**Table 3.** Baseline characteristics of the Whole Life (WL) and comparison group on key demographics and all screening and outcome measures.

Baseline Measure	WL Group ( *n* = 33)	Comparison Group ( *n* = 46)	Test Statistic and *p* value
Gender (m/f)	25/8	32/14	*X ^2^* [1] = 0.367, *p* = 0.55
Age	36.91 (± 10.92)	46.52 (± 11.23)	*t*[77] = -3.8, *p* < 0.001
Length of Illness	11.34 (± 8.82)	20.22 (± 11.75)	*t*[77] = -3.6, *p* < 0.001
PANSS	62.12 (± 12.30)	73.25 (± 18.85)	*t*[77] = -2.95, *p* = 0.004
SASS	29.21 (± 5.24)	28.80 (± 4.60)	*t*[77] = 0.367, *p* = 0.715
SOFAS	54.30 (± 9.86)	45.73 (± 10.81)	*t*[76] = 3.59, *p* < 0.001
HADS	14.15 (± 6.82)	10.61 (± 4.92)	*t*[77] = 2.68, *p* = 0.009
HoNOS	8.15 (± 3.23)	11.67 (± 5.04)	*t*[77] = -3.52, *p* < 0.001
CGI	2.64 (± 0.78)	3.64 (± 1.13)	*t*[76] = -4.4, *p* < 0.001

Table 3 indicates some large differences between the two groups. The distribution of gender is similar for both groups, but the comparison group was significantly older and, accordingly, had a significantly longer duration of schizophrenia (age and duration of illness were significantly, though not perfectly correlated *r* = 0.77, p<0.001), as well as a correspondingly higher total PANSS score (73.25 vs. 62.12). Baseline SASS scores were very similar between groups; imposing a threshold for entry of ≤35 points on the SASS, would likely have had an impact on baseline matching for this measure. On all secondary outcome measures, the comparison group scored significantly higher. Collectively, the data in Table 3 indicate that the comparison group was more ill than the WL group.

### Group comparisons

**Figure 1.**
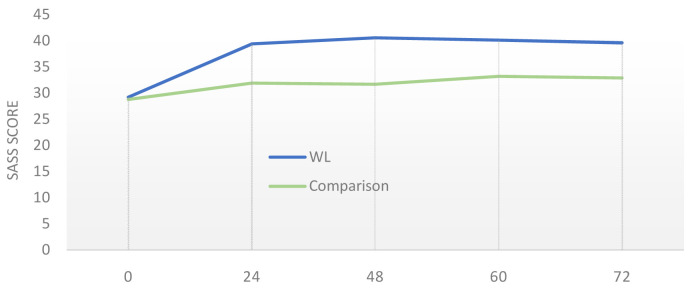
Comparison of WL and comparison across study duration (weeks 0, 24, 48, 60 and 72) on the primary outcome measure (mean SASS score).

Although the comparison group was not a proper control group, it provided an estimate of change in outcome measures under treatment as usual. Differences between the WL and comparison group at week 60 were compared using an ANCOVA model, with PANSS score, age, length of illness, and the relevant baseline score on each outcome measure, entered as co-variates ( Table 4). Although mean SASS score in the WL group had risen from 29.2 to 40.5 by week 60 (vs. 28.8 to 33.7 in the comparison group; Figure 1), this difference was not significant when between-group baseline co-variates were accounted for in the model. The same applied to all secondary outcome measures, with the HADS and HoNOS being the scales closest to showing a borderline significant difference.

**Table 4.** Whole Life (WL) and comparison group scores on primary and secondary outcome measures at baseline and week 60. *F* and *p* values refer to ANCOVA models comparing week 60 scores between groups, with all relevant baseline variables entered as co-variates.

Outcome Measure	WL group ( *n* = 33)				Comparison group ( *n* = 46)				ANCOVA *F*[df], *p*
	Baseline		Week 60		Baseline		Week 60		
	*Mean*	*SD*	*Mean*	*SD*	*Mean*	*SD*	*Mean*	*SD*	
SASS	29.2	5.2	40.5	7.16	28.8	4.6	33.7	8.8	*F*[1,65] = 2.83, *p* = 0.1
HAD	14.2	6.8	12.5	7.5	10.6	4.9	14.5	5.5	*F*[1,65] = 3.80, *p* = 0.06
SOFAS	54.3	9.9	62.8	10.3	45.7	10.8	56.6	10.3	*F*[1,64] = 0.79, *p* = 0.38
CGI-Schiz	2.64	0.78	2.09	0.89	3.64	1.13	2.97	0.97	*F*[1,64] = 0.94, *p* = 0.34
HoNOS	8.15	3.23	6.41	3.91	11.67	5.04	11.41	5.91	*F*[1,65] = 3.67, *p* = 0.06

### Responders

When the 79 study participants were rank ordered, according to degree of improvement on the SASS between Week 0 and 60, the top 10 responders at Week 60 included 8 WL participants, while the lowest 10 (i.e., those whose SASS scores had deteriorated the most) included only participants from the comparison group. It is also notable that one person in the comparison group had a 41-point increase on the SASS between baseline and week 60. While there is no a-priori reason to remove this case, nor treat it as an outlier, it was very uncharacteristic of the comparison group as a whole.

### Relationship between change in outcome measures and key baseline variables

We examined change in each outcome measure, between baseline and week 60, to see whether the level of change was associated with demographic and screening measures recorded at baseline. Turning first to age, this correlated significantly, and negatively, with change in SASS ( *Pearson’s r* = -0.302, *p*=0.007) but did not correlate significantly with any of the secondary outcome measures. Baseline PANSS score also correlated significantly and negatively with change in SASS score ( *Pearson’s r* = -0.318, *p*=0.005) but did not correlate significantly with any of the secondary outcome measures. Participant gender was not associated with change in any outcome measure.

## Discussion

### Contextual issues

This study took place between late 2008 and early 2011, at a time when study treatment costs were still being met by some grant funders, and it long pre-dated the current system for managing excess treatment costs (ETCs) in NHS research. The allocated funding was sufficient to cover provision of the active intervention (WL) only in the host NHS Trust at that time. If the study were run now, the WL programme could have been delivered by any mental health service provider, and the ETCs would have been funded accordingly.

The study was originally conceived as an uncontrolled study, reflecting that: (i) WL had not been formally evaluated in any clinical population previously; (ii) the co-production group were of the view that randomisation could be problematic in this clinical population, at least without a substantive alternative intervention being available; (iii) funding limitations and timelines prohibited a cross-over design. The study was not therefore designed as a randomised control trial (RCT). However, the funding review process requested the introduction of a comparison or treatment-as-usual group, to provide some comparative measures on the key outcome variables. The comparison group turned out to be more severely ill than the WL group at study baseline, and this disparity was factored into all between-group statistical comparisons.

We discuss firstly the feasibility and acceptability of the intervention itself, secondly the between-group comparisons, thirdly the methodological issues and limitations, and finally the possible benefits of using WL or similar approaches.

### Feasibility

Mental health professionals were enthusiastic about using the WL programme and we had no difficulty recruiting coaches for the study. Having some dedicated funding available to create new WL coaching posts, and to backfill time for other participating staff, made this an attractive proposition to managers and their teams at the time the study was run. The majority of WL coaches were mental health support workers, though we also recruited nurses, social workers and psychology assistants. A recurring theme raised by attendees during the training was that WL embodied many of the things that mental health professionals ought to be doing with their service users anyway. There were no instances of coaches dropping out from the study, and feedback from the coaches was generally very positive.

In terms of recruiting service-user participants, the main challenge in this clinical population was to find people who were able, willing and indeed enthusiastic to commit to a year-long programme. It was paramount, therefore, that we asked clinical teams to refer only those people who they felt would benefit from, and be properly committed to, the WL programme. As outlined earlier, schizophrenia is a heterogenous illness and there were many people who were ruled out prior to screening, owing to symptom instability, medication compliance issues, recent hospitalisation or because they were receiving psychotherapy. When we met with people to discuss their participation, we made explicit that the project would entail not just a strong commitment, but also a wish to move forward in their lives, albeit gently and at their own pace. Of the 61 people referred to us for screening, 44 (72%) met inclusion criteria and were thought to be suitable for the programme based upon their intention and enthusiasm to commit. Meeting target recruitment for the study was relatively easy, from which we infer that WL is applicable to a common area of therapeutic concern, widely recognised as such by both clinicians and service users.

Despite the heterogeneity of the underlying illness, there were many things that our participants had in common: most were not in regular employment or education, most felt lonely and had very limited social circles, most had been unable to pursue social activities or hobbies they once enjoyed, most were unhappy with aspects of their physical health and most had low or fluctuating mood. These are all aspects of life that someone with a mental health diagnosis, no matter how severe, should be able to recover, at least to some degree. We could have worked with a different diagnostic grouping, but our aim was to give the WL programme an opportunity to work with a group of people who, arguably, had the greatest need. However, we do acknowledge that although schizophrenia is categorised as a serious mental illness, we were working here with a subgroup of more motivated, and less intractably ill patients.

### Acceptability and retention

The drop-out rate in the WL group was 25%, which matched our initial expectation. Of the 11 who dropped out, the majority found it difficult to commit to a regular meeting schedule and/or to complete the homework exercises between coaching sessions. It is very difficult for the coach and participant to make progress if the homework tasks have been neglected. In general, drop-outs tended to occur quite early on in the programme. A completion rate of 75% is arguably a very positive outcome, given that the WL intervention lasted up to one year, and that the participants were people who had been living with a severe mental illness.

Among the 33 WL participants who completed the study, attendance and adherence to homework tasks were good, with all participants completing the 15 modules. Ratings of perceived benefit of the WL intervention were all positive: no participant indicated that they felt worse at weeks 48, 60 or 72, when compared to baseline. At week 60, 88% of participants said they felt slightly better, much better or very much better, rising to 94% at week 72. Weeks 60 and 72 fell approximately 3 and 6 months after the coaching sessions had ended, so these data suggest that the majority of participants perceived the WL intervention to have more than just a short-term benefit, or one that was only attributable to weekly therapeutic contacts.

Only a small minority (12%) perceived the intervention to have had a neutral effect on how they felt at week 60. Looking at the change in SASS between weeks 60 and 72 ( Figure 1), there is a very slight decrease for the WL group, but this is not statistically discernible.

These data offer support for the feasibility and acceptability of the WL intervention. However, selection of motivated and enthusiastic participants is fundamental to retaining people in the programme. Unfortunately, we were unable to collect longer-term follow up data on any participants, so we don’t know how much the programme assisted them over future years.

### Between group comparisons

Our sample was predominantly male, which is to be expected given the higher prevalence of schizophrenia in men. The gender distribution was similar in the WL and comparison groups. The WL group were generally younger (mean age = 36.9, range 21–59 years) than the comparison group (mean age = 46.5, range 24–65 years) and had correspondingly lower durations of illness, and lower total PANSS scores (mean PANSS for WL = 62.1, range 43–80; mean for comparison group = 73.3, range 37–118; maximum possible range for total PANSS scores = 30–210). So, the comparison group was more chronic and severe than the WL group and, quite possibly, not readily comparable.

These discrepancies had implications for the between-groups analyses. All baseline variables that differed significantly between the groups were entered as co-variates into our model and, on this basis, none of the outcome measures was able to reliably distinguish between the two groups, even though the WL group means at Week 60 were always at a more clinically desirable level. The *p* values for SASS, HAD and HoNOS were all non-significant but fell at a level of 0.1≥ *p*>0.05. This could be indicative of Type II error (the study was only powered at 80%, and this would also assume sample matching, either a-priori, or naturally via randomisation). However, the non-significant group differences are more likely a result of adjustments being made to compensate for the comparison group being more ill. These data suggest that sample size of approx. 50 per group would be adequate in a future study to detect effectiveness but with a strong proviso that adequate sample matching must be ensured.

### Methodological issues and limitations

The comparison group, as configured for this study, represents a major methodological weakness. It would have been our preference to run it as an uncontrolled study, at least initially, to establish proof of principle for WL before proceeding to a RCT. Once preliminary data was available to support acceptability and potential effectiveness, a feasibility RCT would be the preferred methodology to establish the viability of running a larger, fully powered, study.

Our co-production group had voiced concerns that if we introduced randomisation, then patients who had been allocated to the non-active arm would likely withdraw or be non-compliant with research assessments. It does seem likely that retention of control participants would be difficult without offering some kind of incentive. The dropout rate in our comparison group was 39% -- substantially higher than in the WL group. These participants had not been offered nor denied an intervention, so there should have been no element of disappointment for them: they were essentially being asked to take part in a 72-week follow-up study, and the data collection process was less burdensome for them than it was for the WL participants. Nonetheless, the dropout rate was still higher than predicted and could have run even higher if there had been an element of disappointment from not receiving an active intervention. Having adequate control groups for studies of psychological interventions in schizophrenia is an issue that has been highlighted much in recent years (e.g., Laws *et al*., 2018) and one about which there is little current agreement.

Participants could not be blinded to their treatment, and it would be impossible to have blinded the clinical researchers, especially given that the active intervention was only being offered in one location (Hertfordshire). Accepting that it is difficult to eliminate bias where blinding is absent, we favoured a primary outcome measure that was self-rated. Researchers were available to assist with scale completion but, in the main, this was just to check that all items on the SASS and HADS had been fully answered. The remaining (secondary) outcome measures were all scored by the researcher. Several different MHRN staff carried out the clinician-rated outcome measures for all comparison group participants, across three different NHS Trusts, so it seems highly unlikely that there would be any systematic bias to score these participants differently. And indeed, this might have been more of a concern if the between-group analyses had yielded significant differences. Nonetheless, we do acknowledge that blinding of assessors can be a critical factor in studies of CBT in schizophrenia ( Laws *et al.*, 2018), so it seems probable this would apply to other psychological and behavioural interventions too. In larger studies where randomization to more than one treatment group is in place, it would always be preferable to have blinded raters who did not have access to information about the treatment. However this is not something we could implement in the current project, due to its limited scale and scope as a feasibility study.

It is possible that the primary outcome measure we selected was not sensitive enough to distinguish between the groups. However, we doubt this. The week 60 difference between WL and Comparison Group SASS scores was approximately 6 points. This would have been a significant difference (at *p* < 0.01) if there were no baseline group differences to account for, and indeed a 5-point difference is what we based our sample size calculation on. The SASS has 21 items, each rated between 0 and 3, with a maximum total score of 63. A 5- or 6-point increase in SASS score should not be an unrealistic expectation from a one-to-one recovery-focussed intervention spanning 12 months. And indeed, the WL group did improve by approximately 11 SASS points over a 15-month period (a 39% improvement). The SASS also reflects many key themes of personal recovery that are included within the WL manual (e.g., relationships with others, involvement in community life, hobbies, etc.), and was certainly the outcome measure most favoured by our co-production group.

Turning to the HADS, it is a widely used scale for assessing depression and anxiety and has previously demonstrated good reliability and validity ( Bjelland *et al*., 2002). Like the SASS, it would also have differentiated WL and comparison groups based on week 60 scores, were it not for the need adjust for baseline group differences. It should be pointed out that HADS, as a symptom status scale, is a useful supporting measure but has no construct validity directly related to the to the therapeutic objectives of WL.

It seems that HoNOS may be too general a scale to pick up any recovery-oriented improvements, at least in the relatively short-term, and indeed would not have distinguished the groups at Week 60, irrespective of baseline differences. The same is true for the CGI(schiz) and SOFAS scales. CGI(schiz) is, by design, a very brief, global impression scale, and probably not best suited to assessing changes in the chronic features of schizophrenia. However, its parsimony does still make it useful in characterising the group under study.

Finally, the SOFAS covers social and occupational (school or workplace) functioning. The majority of our participants were not in education at the time of the study and the vast majority were not working. Although it would of course be an aspiration of a recovery-focussed intervention to place recipients in a better position to seek employment or further education, we might not expect to see substantial changes in the course of one year, especially when the majority of our participants’ baseline SOFAS scores were well below the threshold used to indicate non-impairment. We were at pains not to over burden participants with too many scales, but in doing so, may have opted for some measures that were too general to detect change. So based on these findings, we would support the use of only the SASS and HADS in any future work on WL and would seek a more sensitive measure of change for educational and occupational activity within this group.

### Is Whole Life useful?

It is notable, when looking at the best (most improved) and poorest (most deteriorated) responders on the SASS, that 80% of the top 10 and 0% of the bottom 10 were WL participants. It is evident, nonetheless, that many comparison group participants made some quite big gains on the SASS. We did not have such detailed information on these people, and it is likely that some of them would have been receiving other psychological interventions. We were unable to rule this out as they were all patients of other NHS Trusts, and we would not have requested that anyone be denied therapeutic input as a result of contributing data to our study. As discussed earlier, this is a further reason why this group is not a proper control for WL. Overall, we did note a significant negative association between age and change in SASS score. Younger people tended to make the biggest gains, but this applied to all participants, not just those in the WL group. A similar association was seen for PANSS; those with lower PANSS scores tended to make the biggest gains in SASS score.

There is certainly no evidence that participating in WL did anyone any harm, and much of our data suggests that it led to noticeable and meaningful improvements for our participants. We would take from all of this, a general view that WL is probably most applicable to younger people: those not having been in the mental health system for too long and who have responded well to anti-psychotic medication, thereby reducing symptom severity. We cannot say that WL is more or less effective than any other type of therapeutic intervention, but we can say that it was well adhered to, and was perceived to be of benefit, by those who took part and completed the full programme.

WL is an example of a recovery-oriented programme. The materials were collected from a variety of sources, most of which are in the public domain. The modules were developed and refined over many years and had significant input from previous recipients of the programme. Although WL is not a manualised therapy per se, the WL Manual is an essential foundation of WL without which, structure and therapeutic coherence could be lost. But no particular materials are, in themselves, 100% essential; and some updating according to social trends would certainly be appropriate. For example, smartphone technology has advanced rapidly in the last decade, so the WL manual might well lend itself towards an app development. Nonetheless, irrespective of the media by which the manual and resources are delivered, the key features of WL are: (i) the coach and participant relationship, with shared responsibility; (ii) the commitment of the participant to engage and be willing to move out of current comfort zones; (iii) the flexible nature of the programme whereby the participant determines pace of work and decides which modules are most helpful to their aims.

We did not undertake any analyses of cost, but we estimate that the WL programme requires approximately 1 to 1.5 hours a week of a mental health professional’s time, for up to a year. Being a WL coach does not require any specific qualifications and is ideally suited to those who are already in a support-worker role.

## Conclusion

In conclusion, we have reported our study findings in relation to both feasibility and effectiveness but have also fully acknowledged the methodological issues present. Nevertheless, it is important that all findings are made available even though the group comparisons did not reach significance. As an intervention, Whole Life is not something that would be applicable to all service users with mental health issues, but it does seem to be a helpful approach when working with those who are committed and motivated to make changes. Moreover, if it can be applied well within a group of people who have had a serious mental illness diagnosis, then it might reasonably be expected to have a useful role in those with less serious, or transitory mental health problems. We would argue therefore, that this NIHR-funded study provides a good basis for undertaking further work on the Whole Life programme

## Data availability

### Underlying data

The original participant consent form, which was approved by the Hertfordshire Ethics Committee, stated that patient data would not be shared outside of the study team. Considering this, the underlying data to the study is not available.

### Extended data

Open Science Framework: Whole Life, https://doi.org/10.17605/OSF.IO/SYGP7 ( Gale, 2021).

This project contains the following extended data:

- A summary of Whole Life programme modules
- Welcome and introduction for Whole Life coaches

Data are available under the terms of the Creative Commons Zero "No rights reserved" data waiver (CC0 1.0 Public domain dedication).

## Author information

Chris J. Hawley is now retired from Hertfordshire Partnership University NHS Foundation Trust, Hatfield, AL10 8YE, UK.
