# Supplementary material for: Whole Life: a feasibility study of a recovery-focussed intervention in patients with stabilised schizophrenia
Source: NIHR Open Res. 2022 Feb 14;1:9. [Version 2] doi: 10.3310/nihropenres.13220.2 (PMC7612533; doi:10.3310/nihropenres.13220.2)
Supplement: Peer review history

# Reviewer report: Reviewer response for version 2

**DOI:** 10.3310/nihropenres.14379.r28485

**Published:** 2022-02-24

**Authors:** Nicole Rose DeTore

**Affiliations:** 1 Harvard Medical School, Boston, MA, USA; 2 Department of Psychiatry, Massachusetts General Hospital, Boston, MA, USA

The additional references to highlight the voices of those with psychosis very much improved the paper, as did the description of the intervention.

Is the work clearly and accurately presented and does it cite the current literature?

Partly

If applicable, is the statistical analysis and its interpretation appropriate?

Yes

Are all the source data underlying the results available to ensure full reproducibility?

Yes

Is the study design appropriate and is the work technically sound?

Yes

Are the conclusions drawn adequately supported by the results?

Partly

Are sufficient details of methods and analysis provided to allow replication by others?

Yes

Reviewer Expertise:

Schizophrenia, recovery, functional outcome, psychosocial intervention

I confirm that I have read this submission and believe that I have an appropriate level of expertise to confirm that it is of an acceptable scientific standard.

# Reviewer report: Reviewer response for version 2

**DOI:** 10.3310/nihropenres.14379.r28487

**Published:** 2022-02-14

**Authors:** Ben Hannigan

**Affiliations:** 1 School of Healthcare Sciences, Cardiff University, Cardiff, UK

My thanks to the team for responding to my comments and queries (and indeed, to those of the other reviewers). I appreciate the revisions which have been made (for instance, relating to the underpinning literature on recovery, and to the origins and the detail of the WL approach). As before, I am not best-placed to say anything about the finer-grained detail of the quantitative techniques used, but with regard to the overall shape and contribution of the paper: I have no further comments to make, and appreciate the work the authors have done here.

Is the work clearly and accurately presented and does it cite the current literature?

Partly

If applicable, is the statistical analysis and its interpretation appropriate?

I cannot comment. A qualified statistician is required.

Are all the source data underlying the results available to ensure full reproducibility?

No

Is the study design appropriate and is the work technically sound?

Yes

Are the conclusions drawn adequately supported by the results?

Yes

Are sufficient details of methods and analysis provided to allow replication by others?

Partly

Reviewer Expertise:

I am a mental health systems and services researcher with a primary background in in-depth qualitative methods. I do not have expertise in statistical analysis or reviewing.

I confirm that I have read this submission and believe that I have an appropriate level of expertise to confirm that it is of an acceptable scientific standard.

# Reviewer report: Reviewer response for version 1

**DOI:** 10.3310/nihropenres.14336.r28449

**Published:** 2021-12-13

**Authors:** Nicole Rose DeTore

**Affiliations:** 1 Harvard Medical School, Boston, MA, USA; 2 Department of Psychiatry, Massachusetts General Hospital, Boston, MA, USA

This paper does a fantastic job of presenting an important type of treatment aimed at improving recovery within mental illness. It well presents the study design in an upfront and open manner which allows the reader to make appropriate inferences.

 As a reader I was left with a few questions/ comments that I will describe below:

- It sounds as though the 15 modules are paced across one year in a way that best fits the needs of the participant - however is there any information on the average number of sessions obtained by participants? Just to get an idea of the length of the WL intervention.
- The demographics are presented as a comparison between the two groups, but I do not see any information on the racial/ethic make-up of the sample which would be helpful to know.
- Similarly, work or school status is often a characteristic that is nice to know when thinking about recovery - both their current work/school status as well as their past work/school history. If those data are available, it would be nice to know as the reader.
- There are some places in the introduction and description of schizophrenia section where statements are made that describe how some people with schizophrenia feel. It might be nice to include a citation of those ideas so that as a reader, we could see more literature about those feeling specifically. It would also be nice to see that these assumptions are made based off research where those with schizophrenia either stated it qualitatively or endorsed it quantitatively. This includes the statement that people with schizophrenia are cautious to leave home for fear of a trigger. There are many reasons some with psychosis do not work, so either a citation of that idea or citations of other ideas would better support it.
- The SASS is not a measure I commonly see in schizophrenia literature and seems more based out of the depression literature. Including a citation of it's use in schizophrenia could help the reader learn more about the measure itself in this population.
- Not until the discussion do you mention that these WL sessions could be held in the community. It is also not spelled out where it could be held (in one's home, coffee shop, etc.). It would be helpful as a reader to understand the ways in which this was delivered to meet the needs of clients- also to assess it's feasibility. 
- My last two comments are regarding the SOFAS. I found myself wondering why the SOFAS was not explicitly stated in the 'comparison between groups' section of the paper. It looks as though it was significantly different (p=.03) between the two groups, with the WL group scoring higher. That difference feels important to me when contextualizing the comparison group as being more 'chronic and severe'. Symptomatically, but possibly not in functioning - which when using a recovery lens, is really the most important qualifier - beyond symptoms on the PANSS. The SASS was also higher in the WL group, therefore the comparison group may have been more chronic, but I wonder if a more specific word could be chosen than just 'severe' as I think it could be argued that is not the case.
- In the discussion, authors mention that it is not expected to see work/school goals change over the one year period (in reference to the SOFAS). Although, a weekly, year long intervention targeting recovery in SMI is, often in research, deemed appropriate to obtain work/school outcomes. This was not an entirely employment focused intervention like supported employment, etc., however, it does have at least one module dedicated solely to work/school and many focused on goal setting and planning for their future life. I would therefore question the idea that the SOFAS could not change across a one year intervention and that it may not be entirely aspirational.

Is the work clearly and accurately presented and does it cite the current literature?

Partly

If applicable, is the statistical analysis and its interpretation appropriate?

Yes

Are all the source data underlying the results available to ensure full reproducibility?

Yes

Is the study design appropriate and is the work technically sound?

Yes

Are the conclusions drawn adequately supported by the results?

Partly

Are sufficient details of methods and analysis provided to allow replication by others?

Yes

Reviewer Expertise:

Schizophrenia, recovery, functional outcome, psychosocial intervention

I confirm that I have read this submission and believe that I have an appropriate level of expertise to confirm that it is of an acceptable scientific standard, however I have significant reservations, as outlined above.

# Response

**Published:** 2022-02-08

**Authors:** Tim Gale

**Affiliations:** Hertfordshire Partnership University NHS Foundation Trust, UK

Thank you. Yes we do have some data on contact time. A similar point was raised by R2 and we have now added an additional paragraph in the first section of the Results (entitled Recruitment and Retention), which covers the frequency and duration of meetings between coach and participant.

 Unfortunately we did not log ethnicity details. We realise that almost all research would record ethnicity information these days, but when we ran the study, there was less emphasis on this. We know that the sample was predominantly Caucasian, though not exclusively. However, regrettably, we cannot provide a precise breakdown.

 We have added two references to support our comments about comfort zones, including an article written by someone who has experienced schizophrenia first-hand. The notion of a ‘comfort zone’ is quite widely reported, along with the need for people with schizophrenia to gradually expand this on route to regaining some of their life.

 It is true that the SASS has primarily been used in depression studies but it has sometimes been used in studies of people with schizophrenia. We have added two references to support this (last paragraph of Introduction, under ‘Study Aims’ sub-heading).

 Thank-you for this suggestion about location of WL meetings. Flexibility is at the heart of WL, and it is not prescriptive about where people meet their coach. We have added some additional text to this effect in the Introduction under the sub-heading ‘The Whole Life Programme’ (3 ^rd^ para).

 I am unsure where a p=.03 difference is shown in relation to the SOFAS. I can’t see this in any of the text or figures. The SOFAS is included in table 3, which was intended to highlight the baseline differences between the WL and Comparison groups. Since there was a significant baseline difference, we employed an analysis of covariance (ANCOVA) model to compare this data, which takes into account the pre-existing baseline difference. Under this analysis there was no significant difference between groups. The WL group went from 54.3 to 62.8, while the Comparison group went from 45.7 to 56.6 at week 60 (Table 4). Since both groups’ score increased over the study duration, there was no significant difference and we did not discuss it further, apart from mentioning that we would probably drop this scale in favour of something more sensitive if future work on WL were to be carried out.

 We agree with you about expectation of educational/occupational change in response to a year long intervention programme. Indeed, we did see an increase on the SOFAS score, albeit small. However, as outlined before, SOFAS score also increased in the Comparison (no intervention) group so it may lack the sensitivity needed to detect change in a relatively impaired group of people. We have added some text in the discussion (last para of section entitled ‘Methodological Issues and Limitations’ 

 Thank you Nicole for taking the time to read and review our paper.

# Reviewer report: Reviewer response for version 1

**DOI:** 10.3310/nihropenres.14336.r28429

**Published:** 2021-11-24

**Authors:** Helen Louise Brooks

**Affiliations:** 1 Division of Nursing, Midwifery and Social Work, University of Manchester, Manchester, UK

Thank you for asking me to review this manuscript which reports on the feasibility of implementing a recovery-focussed intervention for patients with schizophrenia. I thought the manuscript was well written and the topic is likely to be of interest to a wide audience.

**Introduction:**

- This gave a useful overview of the study but I felt a more critical discussion of the concept of recovery and its application in the mental health field would have strengthened this section.
- The introduction states that this is the first time the intervention has been the subject of research testing but later refers to the manual being refined over many years by the participants who have used it. Clarification here would be helpful.
- More information on the development of the WL programme would also have been useful as the current level detail is insufficient. In particular, the co-productive aspects that are referred to would be a welcome addition. A description of the intervention itself using the TIDieR framework would also be helpful perhaps as a supplementary table.

**Methods: **

- Could the term standardised schizophrenia be defined?
- The study appears to be have completed some time ago.  Could the authors provide justification for this?
- How were participants matched with health professionals?
- Was there a minimum level of engagement with the intervention that was considered sufficient?

**Results:**

- What were the reasons participants gave for not being able to commit to the regular meetings and completion of homework?
- I have not commented on the statistical analysis and this element would require input from a statistician.

**Discussion: **

- The first paragraph in the feasibility section refers to qualitative analysis which doesn't appear to be reported in the manuscript itself. Could this be referenced?
- Could the use of professional gatekeepers be discussed in light of potential bias and future implementation of the intervention.
- Were success criteria in terms of key feasibility areas (e.g. drop-out rates) agreed before the study started?
- It would be more helpful to use the term effectiveness consistently rather than the interchangeable use of effectiveness and efficacy.

Thank you again for inviting me to review this manuscript.

Is the work clearly and accurately presented and does it cite the current literature?

Yes

If applicable, is the statistical analysis and its interpretation appropriate?

I cannot comment. A qualified statistician is required.

Are all the source data underlying the results available to ensure full reproducibility?

Partly

Is the study design appropriate and is the work technically sound?

Yes

Are the conclusions drawn adequately supported by the results?

Partly

Are sufficient details of methods and analysis provided to allow replication by others?

Partly

Reviewer Expertise:

Qualitative, co-production and implementation science.

I confirm that I have read this submission and believe that I have an appropriate level of expertise to confirm that it is of an acceptable scientific standard, however I have significant reservations, as outlined above.

# Response

**Published:** 2022-02-08

**Authors:** Tim Gale

**Affiliations:** Hertfordshire Partnership University NHS Foundation Trust, UK

Thank you. We have added some additional text at the end of the section entitled ‘Recovery approach in mental health care’ to point out the tension between recovery approaches and medical models of care, and that they are probably most optimally used in conjunction.

 We have added additional text in paragraph 3 under ‘The Whole Life programme’ heading, which hopefully clarifies that the WL programme was developed over several years before the final version became the focus of a feasibility study. We have expanded the section on the co-production aspects, as well as the rationale and relationship with learned helplessness. And we have also made it clearer that the WL programme is not overly prescriptive about timing, location or sequence of modules. We did submit a CONSORT table with the paper but did not include a description within the TIDieR framework. I can see the benefit of a TIDieR framework in pinning down exactly what an intervention entails, but it seems to be something that is more easily applied to medical models, whereas WL embodies flexibility and person-centred intervention so it is quite difficult to accurately capture it within such a framework. However if the reviewers and editorial team would like us to submit one, we will of course do this.

 Unless I have missed something, we did not used the term ‘standardised schizophrenia’. We did however use the term ‘stabilised schizophrenia’, by which we meant that the participant was compliant with treatment and had not been recently hospitalised. We have now added a note to refer the reader to the next section (Participants: inclusion and exclusion), which quantifies what is meant by ‘stabilised’.

 The study was indeed completed some time ago, as also pointed out by reviewer 1 (please see also our response to R1). We acknowledge that such a long lag between carrying out the research and reporting it is far from ideal, but this was partly due to factors outside our control and we do think it is important that the findings are made publicly available nonetheless.

 Participants were matched with health professionals primarily by geographical location. There was no randomisation here as we simply sought to make the arrangements as easy for participant and coach as possible. We have added clarification about this under the ‘Participants: coaches’ heading.

 We did measure the number of contact hours between coach and participant, and we have now added an additional paragraph under ‘Recruitment and retention’ at the start of the results section. We tried to be flexible and allow participants to remain in the study unless they (i) opted out or (ii) failed to meet with their coach for more than a month, and we did find that where a month or more had elapsed, the participants generally no longer wanted to engage. However, a key feature of WL is that it permits flexibility, so we did not set a minimum level of engagement.

 We did not collect data on the reasons people gave for withdrawing. In most cases this was conveyed by the service user to the coach, who then reported it back to the central study team. The general pattern seemed to be that some people just found it difficult to keep to the weekly meetings and then became less communicative with their coaches. Once this happened, the coach would usually ask the service user if they would prefer to withdraw. There is a qualitative paper (Littlechild *et al*. 2014) which does report in greater depth about the individual participant experiences within our cohort, and we have referenced this at the end of the ‘Data collection and outcome measures’ section of the Method.

 We are a bit unsure how gatekeepers would eliminate bias as most studies of psychological intervention would require some face-to-face contact between participant and research assessor (unless all outcome measures were self-rated). We would certainly agree that rater blinding is always preferable when there is more than one treatment condition, and have added additional text to this effect.

 The key feasibility areas are outlined in general terms under Study Aims, but we did not quantify these in terms of percentages that would indicate success or failure. We acknowledge that the requirements for feasibility studies are more rigorously defined nowadays, but at the time we applied for funding for this work, we were not requested to do this. We have replaced ‘efficacy’ with ‘effectiveness’ throughout the manuscript. Thank you for taking the time to read and review our paper Helen.

# Reviewer report: Reviewer response for version 1

**DOI:** 10.3310/nihropenres.14336.r28436

**Published:** 2021-10-01

**Authors:** Ben Hannigan

**Affiliations:** 1 School of Healthcare Sciences, Cardiff University, Cardiff, UK

Thank you for this opportunity to read and review this paper for NIHR Open Research. I see that this is a new way of sharing scholarly work, involving post-publication peer review: an interesting initiative, I think.

 This paper is well-written, and reports on the feasibility and acceptability of the Whole Life coaching programme. This is something that is new to me and is described as an intervention that aims to promote recovery with people living with severe mental health problems.

 I'm struck, in the first instance, on the age of the data reported here: is there a reason why this study completed its data generation a decade ago? I see that the qualitative component was reported seven years ago, too. I do not see this time lag as a major problem, but it does make me somewhat curious (and also makes me ask questions on the further rolling-out of the WL approach in the time since 2011). Where there is, I think, a definite time-related issue is with the citing of sources on the concept of 'recovery'. These appear at the paper's opening, and could do with an update: particularly in the context of the claim that 'over the last few decades...'. I suspect the policy and evidence context for recovery approaches has moved on, and this might usefully be acknowledged.

 The WL programme is introduced at the start of the paper, with support from a more expansive account included as a supplementary file. Although hinted at throughout the manuscript I cannot, quite, find a section comprehensively reporting on how the approach was developed and co-produced. I appreciate the reference to its sensitivity to feedback from people who have participated in the past (under the heading 'co-production'), but to me, this seems to fall short of the kind of intervention development description that I had hoped for. How, for example, has the intervention been informed by the theory of learned helplessness? How were the modules conceived and sequenced? What are the conjectured mechanisms of effect, as it were? The supplementary file, I think, does not quite help here as although it (usefully) expands on the modules it has less to say on the programme’s origins and development.

 All this said the WL approach is interesting in trying to operationalise some of the principles of recovery-oriented practice. So far as I can tell the WL programme has a high degree of flexibility, and service users and practitioners are (were?) able to engage over a protracted period.

 With regards to research process issues: here, I can see how tensions arose between the wishes of service user advisors and the demands of the research funder (as an aside: is the funder acknowledged anywhere?). This clearly caused difficulties with study design and methods, and the initially unplanned introduction of a comparison group. Thinking, too, about the coaches (whose involvement would have been so critical as it was entirely through them that the intervention lived and breathed): has the team any reflections on the training for coaches, given that this was limited to one day only? I can see how updates and supervision were ongoing, but one day as preparation seems rather minimal.

 I think the account of how outcome measures were selected is fine, and I am reminded of the purpose of this study as being a feasibility and acceptability one and not to definitely evaluate effectiveness. The estimates of sample size and the statistical tests I'll definitely leave to those more qualified in this area to comment on. Rates of participation and major findings are clearly reported, though, along with an account of participants' experiences and views of the coaching programme. I think the conclusions drawn on the suitability of the programme for groups of service users, and the selection of outcome measures in the future, are appropriately tentative.

 Overwhelmingly I am, though, interested to know how the lessons learned in this experience have been accommodated in further iterations of the WL approach in the years following its introduction in this study.

 I hope this review is helpful to the team, and invite their response.

Is the work clearly and accurately presented and does it cite the current literature?

Partly

If applicable, is the statistical analysis and its interpretation appropriate?

I cannot comment. A qualified statistician is required.

Are all the source data underlying the results available to ensure full reproducibility?

No

Is the study design appropriate and is the work technically sound?

Yes

Are the conclusions drawn adequately supported by the results?

Yes

Are sufficient details of methods and analysis provided to allow replication by others?

Partly

Reviewer Expertise:

I am a mental health systems and services researcher with a primary background in in-depth qualitative methods. I do not have expertise in statistical analysis or reviewing.

I confirm that I have read this submission and believe that I have an appropriate level of expertise to confirm that it is of an acceptable scientific standard, however I have significant reservations, as outlined above.

# Response

**Published:** 2022-02-08

**Authors:** Tim Gale

**Affiliations:** Hertfordshire Partnership University NHS Foundation Trust, UK

Thankyou. There are personal reasons behind the delay in publishing the findings, and I would prefer not to elaborate on these. Whole Life was very popular with those people who took on the coaching role within the study, and it is still in use today although we have not carried out further research on it. The reason that the qualitative study was published much sooner is because it was a separate module, run primarily by a different group, and its completion was not contingent on seeing any of the quantitative data.

 Much of the recovery literature is more than a decade old, and this is partly because the concept of recovery seems to have become so well engrained in mental health policy and practice in more recent times. We have, however, added additional references to two more recent texts from Mike Slade’s group, and a more recent paper on service user perspective around personal recovery journeys (see Introduction, para 1). We have tried to clarify the evolution of the WL manual itself, which grew to accommodate the expressed needs of people who were using it as part of their own journeys. This was not part of the research study, and so there is no documentation on this. Some additional text is provided under the ‘Co-Production’ heading. In the section entitled ‘Whole Life’ we have expanded on the relationship between learned helplessness and the WL manual, and also the proposed mechanism of effect.

 The funder was NIHR and has been duly acknowledged under the section ‘Grant Information’ which appears below the abstract.

 The training for WL coaches was indeed a single day, but they received regular supervision sessions after this and could also contact the WL author (JW) at any time for additional support. None of the material in the WL manual is conceptually difficult, so the training focussed on the principles of recovery, and ways in which the service user could be supported to take control, rather than the health professional doing the work for them. It included some role play scenarios. Regular supervision allowed the coaches to seek clarification on any module content if required, although it was our experience that most found it very straightforward.

 Thank you very much for your thoughtful comments Ben. Unfortunately we have not been able to carry out further research on WL, though it is possible that this publication might stimulate further interest. If we did a further study, we would probably focus on a population with less serious mental illness, as we believe that the approach may have a stronger and more lasting effect. The WL manual is still quite widely used by support workers and teams in our Trust, but unfortunately we do not have any hard data beyond what has been reported in this paper.
